# Biomechanical efficacy of AP, PA lag screws and posterior plating for fixation of posterior malleolar fractures: a three dimensional finite element study

**DOI:** 10.1186/s12891-018-1989-7

**Published:** 2018-03-06

**Authors:** Adeel Anwar, Zhen Zhang, Decheng Lv, Gang Lv, Zhi Zhao, Yanfeng Wang, Yue Cai, Wasim Qasim, Muhammad Umar Nazir, Ming Lu

**Affiliations:** 1 Department of Orthopaedic Surgery, The First Affiliated Hospital of Dalian Medical University, 222 Zhongshan road, 116011 Dalian, Liaoning People’s Republic of China; 2 Department of Orthopaedic Surgery, The First Affiliated Hospital of China Medical University, 155 Nanjing north street, 110001 Shenyang, Liaoning People’s Republic of China; 3 Department of Orthopaedic Surgery, The Second Affiliated Hospital of Dalian Medical University, 456 Zhongshan road, 116027 Dalian, Liaoning People’s Republic of China; 4 Department of Automation, School of Electrical Engineering, Dalian Jiaotong University, 794 Huanghe road, 116028 Dalian, Liaoning People’s Republic of China; 5 Department of General Surgery, The Second Affiliated Hospital of Dalian Medical University, 456 Zhongshan road, 116027 Dalian, Liaoning People’s Republic of China; 6 Department of Respiratory Medicine, The Second Affiliated Hospital of Dalian Medical University, 456 Zhongshan road, 116027 Dalian, Liaoning People’s Republic of China

**Keywords:** Posterior malleolar fracture, Fixation, Biomechanical, Three dimensional, Finite element analysis

## Abstract

**Background:**

Clinically there are different fixation methods used for fixation of the posterior malleolar fractures (PMF), but the best treatment modality is still not clear. Few studies have concentrated on this issue, least of all using a biomechanical comparison. The purpose of this study was to carry out a computational comparative biomechanics of three different commonly used fixation constructs for the fixation of PMF by finite element analysis (FEA).

**Methods:**

Computed tomography (CT) images were used to reconstruct three dimensional (3D) model of the tibia. Computer aided design (CAD) software was used to design 3D models of PMF. Finally, 3D models of PMF fixed with two antero-posterior (AP) lag screws, two postero-anterior (PA) lag screws and posterior plate were simulated through computational processing. Simulated loads of 500 N, 1000 N and 1500 N were applied to the PMF and proximal ends of the models were fixed in all degrees of freedom. Output results representing the model von Mises stress, relative fracture micro-motion and vertical displacement of the fracture fragment were analyzed.

**Results:**

The mean vertical displacement value in the posterior plate group (0.52 mm) was lower than AP (0.68 mm) and PA (0.69 mm) lag groups. Statistically significant low amount of the relative micro-motion (*P* < 0.05) was observed in the posterior plate group.

**Conclusions:**

It was concluded that the posterior plate is biomechanically the most stable fixation method for fixation of PMF.

## Background

The posterior malleolar fractures account for 7%–44% among the ankle fractures [1–5]. Ankle fractures involving the posterior malleolus have worse prognostic outcomes compared with ankle injuries without involvement of the posterior malleolus [1]. The treatment recommendations for the medial and lateral malleolar fractures are well recognized, but the guidelines for surgical fixations used for posterior malleolar fractures remain unclear [6–8], and there is an ongoing debate on this issue. Though most of the researchers agreed that the posterior malleolar fractures of > 25% articular involvement should be fixed surgically, but several studies have argued the importance of smaller size fractures and evaluated the critical role of posterior malleolus fixation for syndesmotic stabilization [9–14]. Different surgeons approach differently to treat the posterior malleolar fractures. By using the indirect anterior approach, it can be reduced through ligamentotaxis and fixation can be achieved by antero-posterior (AP) lag screws. Conversely, fracture can be reduced by using direct approach (postero-lateral), in which internal fixation is maintained either by postero-anterior (PA) lag screws or by posterior plating. Less attention has focused on the prognosis of the different fixation constructs. A sound knowledge of comparative biomechanical efficiency of the different fixation constructs is essential for trauma surgeons to choose the optimal fixation method in ankle fractures involving the posterior malleolus and for improved clinical outcomes. Highly variable fracture patterns of the posterior malleolar fractures were classified by Haraguchi et al. [15] into three distinct categories. Among these three types, the type 2 (29.8% of the tibial plafond involvement) meet the current fixation indications. The aim of this study was to evaluate the biomechanical efficiency of AP lag screws, PA lag screws and posterior buttress plate for the fixation of posterior malleolar fractures of > 25% size. This size was selected because at present most of the trauma surgeons are agreed to fix the fractures of more than 25% size. Finite element analysis (FEA) was conducted to compare the stress and displacement patterns in different load conditions.

## Methods

### Three dimensional (3D) modeling

**Fig. 1:**
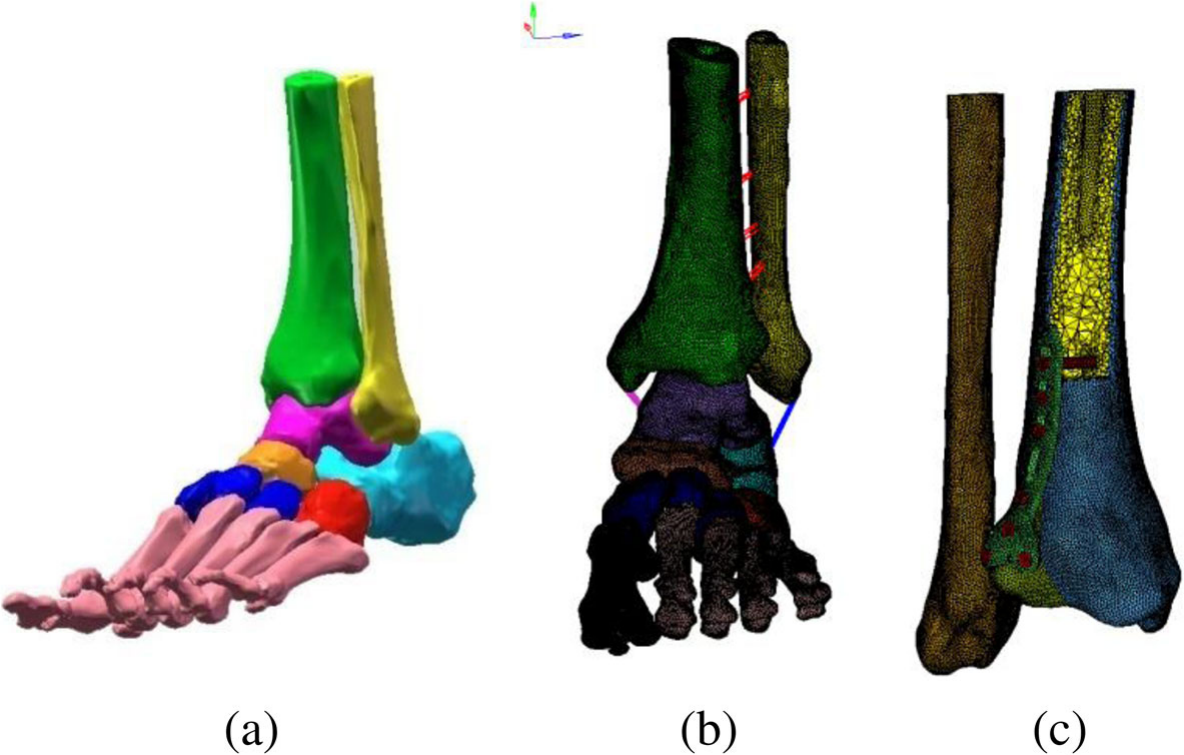
3D ankle model (**a**) Initial 3D modeling, (**b**) Meshing of the processed model, (**c**) Finite element model showing cortical and cancellous portions of the bone

**Fig. 2:**
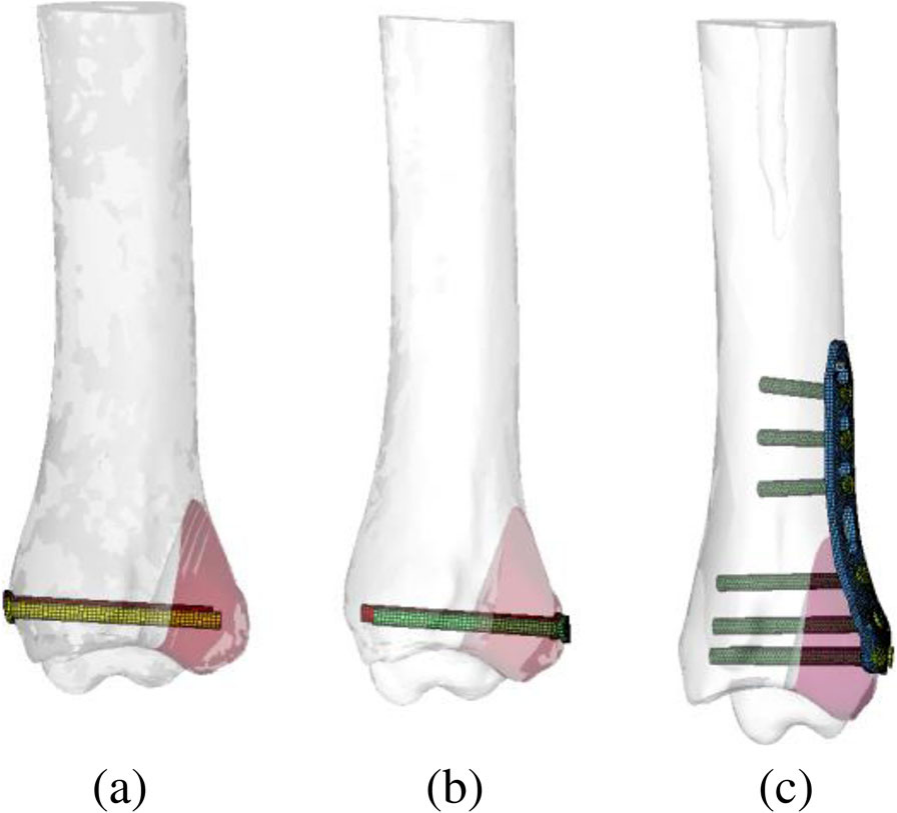
Model of posterior malleolar fracture showing three different fixation strategies. **a** Two AP lag screws, **b** Two PA lag screws, **c** Posterior plate

CT scan images of the right ankle of a volunteer in the neutral unloaded position were used to reconstruct the three dimensional (3D) model of the ankle joint. There was no past history of trauma or anatomical abnormality. This research work was approved and in accordance with the Medical Research Ethics Committee of the author’s hospital. Experimental work was performed in accordance with the Helsinki Declaration. CT scan data in Dicom form was then imported into Mimics 10.1 software (Materialise, Leuven, Belgium) to reconstruct the surface geometry of the bones. Then structure of the each bone in igs format was transferred to Geomagics 11.0 software (Raindrop Company, USA). Processing in Geomagics was done to obtain the volumes of the bones. Stp files of bone’s volume were imported into Pro E (CREO 3.0 PTC Corp., USA). Finally, the solid objects representing the bones were assembled within Pro E to make 3D foot-ankle complex (Fig. 1 a). The coordination axes of the 3D assembled model were assigned as; X-axis pointed posteriorly (toe to heel), Y-axis pointed upward (heel to knee) and Z-axis pointed laterally (medial to lateral malleolus). To obtain the geometry of posterior malleolar fracture of > 25% size, the postero-lateral margin of the ankle was modeled in Pro E Fig. 1 (c). In this study, an exact fit fracture model was illustrated; there was no fracture gap between the fracture fragment and the remaining bone. However, the posterior fragment can move in relation to the un-fractured bone with an assigned friction coefficient of 0.3 [16]. Three different fixation modalities; two AP lag screws, two PA lag screws and posterior malleolar anatomic plate were adapted to fix the fracture models as shown in Fig. 2. The 3D models of the implants were designed using the computer aided design (CAD) software (ProE CREO 3.0 PTC Corp., USA)). All the assembled models were meshed using the HyperMesh 11.0 software (Altair Engineering, Inc., USA). Fig. 1(b).

### Finite element (FE) modeling and material properties

3D models of tibia with associated fixation implants in ing files were then imported into finite element analysis software Abaqus 6.14 (Simulia Corp., USA). In Abaqus, interaction between different parts of the models was performed. Bone was assumed to behave as homogeneous, isotropic and linearly elastic material. The cortical and cancellous portions of the distal tibia were modeled with Young’s modulus (E) of 7300 and11100 MPa and Poisson ratio (y) of 0.3 and 0.26 respectively [17, 18]. Fixation implants including screws and plate were assigned an elastic modulus and Poisson’s ratio of 110,000 MPa and 0.3 respectively [19]. The effect of gravity was considered as negligible in the model. Material properties are given in Table 1.

**Table 1:** Material properties used in finite element models

Material	Young’s modulus (E)	Poisson ratio (y)	References
Cortical bone	7300	0.3	[17]
Cancellous bone	1100	0.26	[18]
Fixation constructs	110,000	0.3	[19]

### Boundary conditions and validation of model

**Fig. 3:**
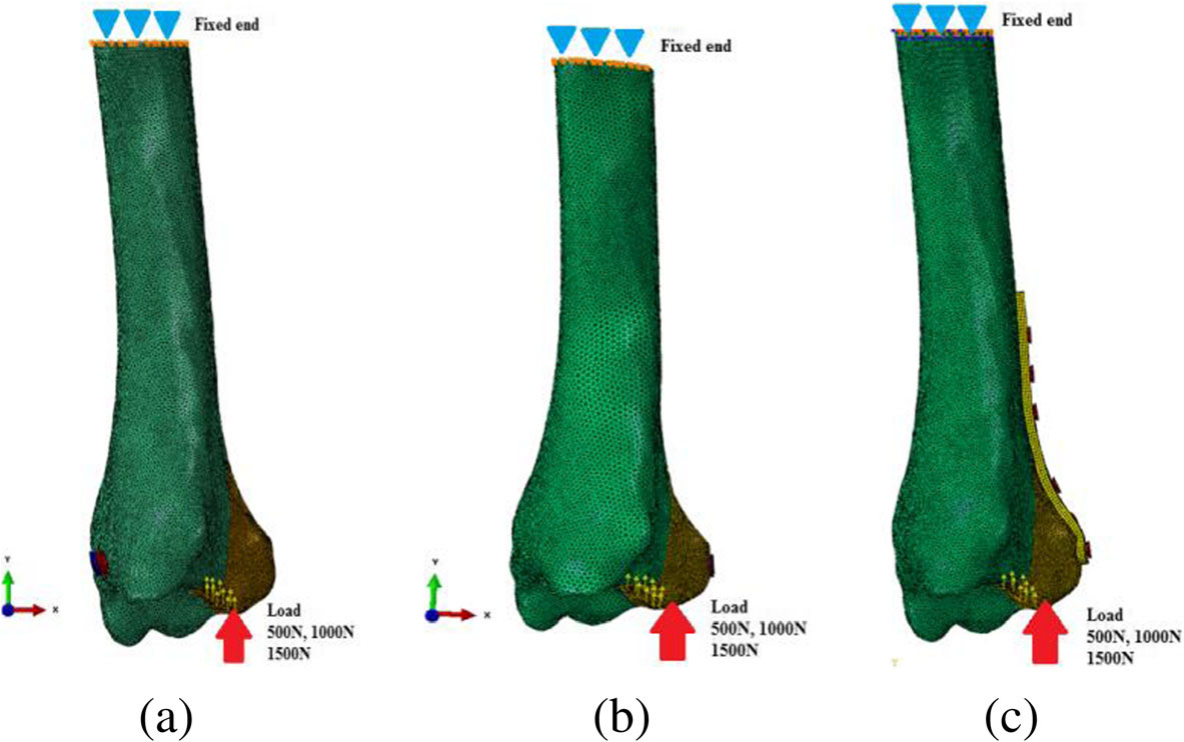
Finite element model showing boundary conditions and load direction. **a** AP lag group, **b** PA lag group, **c** Plate group

3D model is validated as it has cortical shell and inner cancellous bone and material properties were assigned accordingly [17, 18]. The proximal end of the tibia was fixed in all degrees of freedom as shown in Fig. 3. In this experiment, the force was applied to the fracture fragment in the direction of fracture mechanism [20]. Three different magnitudes of axial force i.e.; 500 N, 1000 N and 1500 N were simulated according to previous study [21]. The analysis was done using finite element software Abaqus 6.14 (Simulia Corp., USA). In put units of KPa, seconds and mm were used for elastic modulus, time and displacement in this analysis respectively. Von Mises stress output unit was KPa, and final stress values are represented using MPa. For easy interpretation of the results, we divided the 3D models in three groups; model using two lag screws in antero-posterior direction was assigned as AP lag group and models with postero-anterior screws and posterior plate were named PA lag and plate groups respectively as shown in Fig. 3.

### Statistics

Statistical analysis was done using SPSS 16.0 (SPSS Inc., Chicago IL). Descriptive statistics were used to determine means and standard deviations. One way ANOVA (Post Hoc/LSD) was used for multi comparisons.

## Results

The total number of nodes were 71,970, 57,342 and 85,825 in AP, PA and plate models respectively and total number of elements were 333,768, 273,181 and 412,913 for AP, PA and plate respectively.

### Von Mises stress (VMS) patterns

**Fig. 4:**
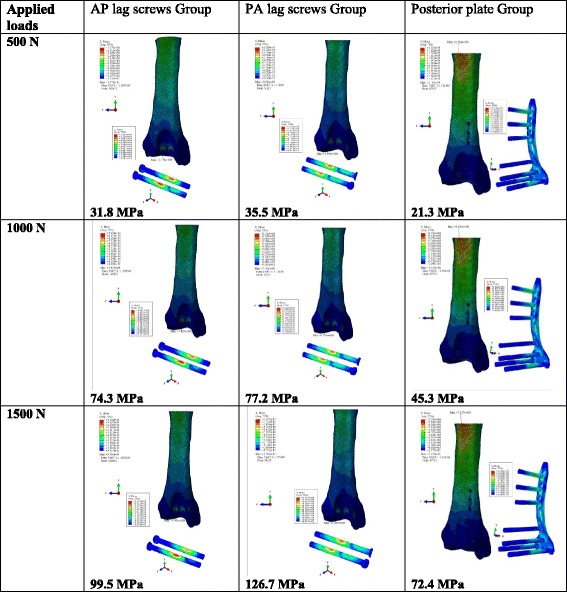
Von Mises Stress (VMS) pattrens in three models with loads of three different magnitudes

**Fig. 5:**
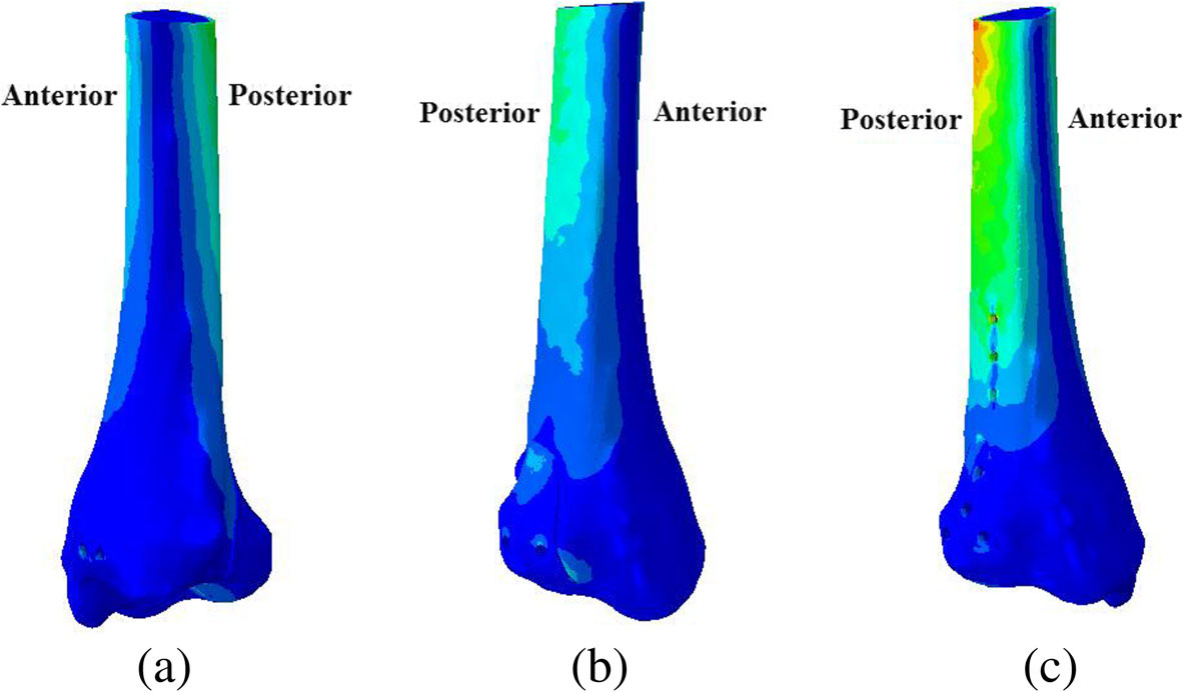
Peak von Mises Stress (VMS) stress distribution in models using three different fixation strategies. **a** AP, **b** PA, **c** Posterior plate models

Von Mises Stress (VMS) distributions in the three models using different magnitudes of the load are shown in Fig. 4. Over all stress patterns showed more concentrated stress in upper anterior and posterior portions of tibia (Fig. 5). The bone model with AP lag screws showed the highest peak values. Higher stress values were observed in AP and PA lag screws. The stress distribution was concentrated in the screw area near the fracture line. In plate group stress areas were also observed in the upper three screws and plate junctions.

### Model displacement

**Fig. 6:**
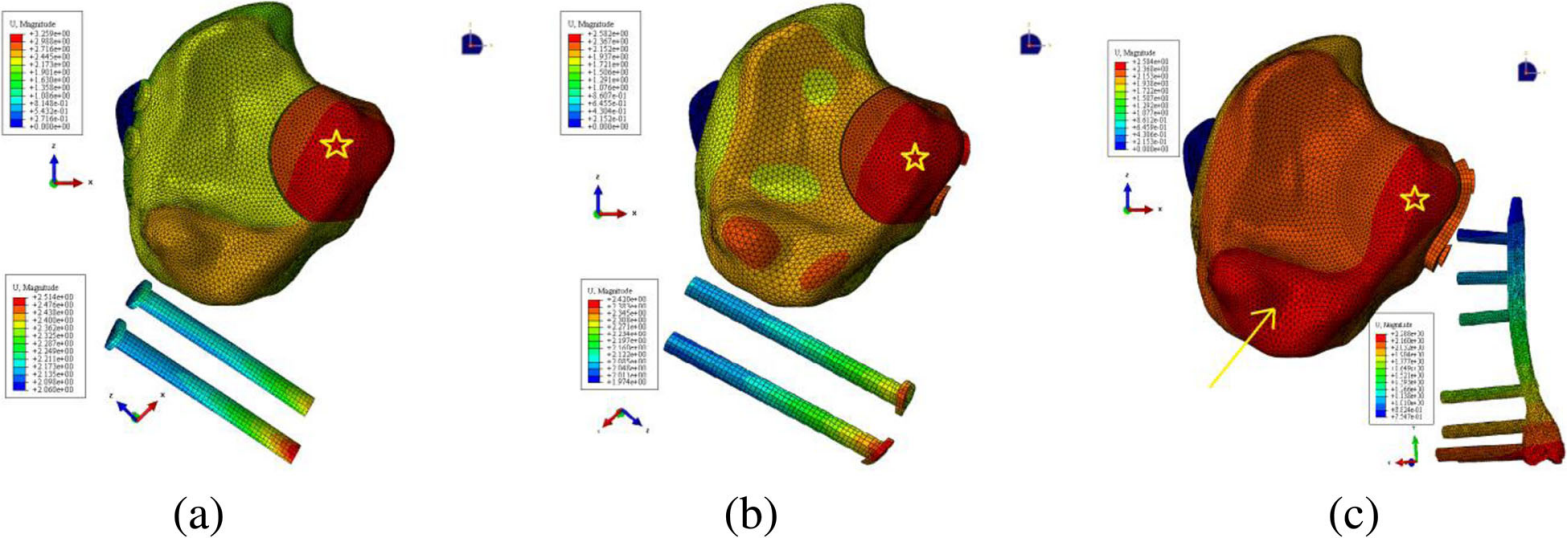
Tibial plafond showing the model and implant displacements (mm). Star represents the displacemnt of fracture fragment only. Arrow represnts the movement of medial malleolus. **a** AP lag, **b** PA lag, **c** Posterior plate models

**Fig. 7:**
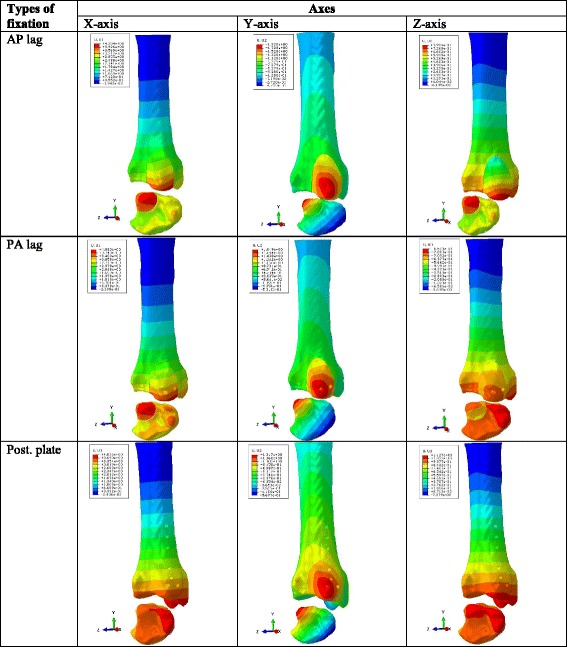
Representation of displacement patterns in X,Y and Z axes in the higest load group (1500 N)

Analysis of the model displacement shows that AP lag group shows the highest amount of displacement. Whereas, PA lag and plate groups show nearly same amount of displacement. In AP and PA lag models, mainly the fracture fragment is displaced, but displacement is also prominent in the tibial plafond in posterior plate model (See Fig. 6). In X-axis (posterior component of displacement), the highest displacement was observed in AP lag model (1.27, 3.12, 4.28 mm). PA lag model with displacement values of 1.12, 2.45, 4.08 mm shows better fixation power than AP group. The posterior plate group had slight higher displacement values (4.45, 4.29, 4.19 mm). Y-axis represents the vertical displacement in direction of the applied load. This vertical movement is higher in AP group followed by PA and plate groups. The largest VD values were noted in the 1500 N loading. In this loading condition, PA lag showed 1.46 times more VD than posterior plating and PA lag showed 1.4 times higher displacement than plate group. Key movement of fracture in three co-ordinate axes using 1500 N compressive force is given in Fig. 7. Z-axis (lateral movement), plate shows the greater amount of distal tibia. Table 2 shows the displacement values in three axes.

**Table 2:** Displacement values (mm) in X, Y and Z axes

Loads	Fixation methods	Axes		
		X-axis	Y-axis	Z-axis
500 N	AP lag screw	1.27	0.57	0.26
	PA lag screw	1.12	0.52	0.23
	Posterior plate	1.16	0.37	0.30
1000 N	AP lag screw	3.12	1.40	0.61
	PA lag screw	2.45	1.14	0.51
	Posterior plate	2.49	0.86	0.67
1500 N	AP lag screw	4.28	1.93	0.81
	PA lag screw	4.08	1.85	0.85
	Posterior plate	4.03	1.32	1.13

### Relative micro-motion (RM)

**Fig. 8:**
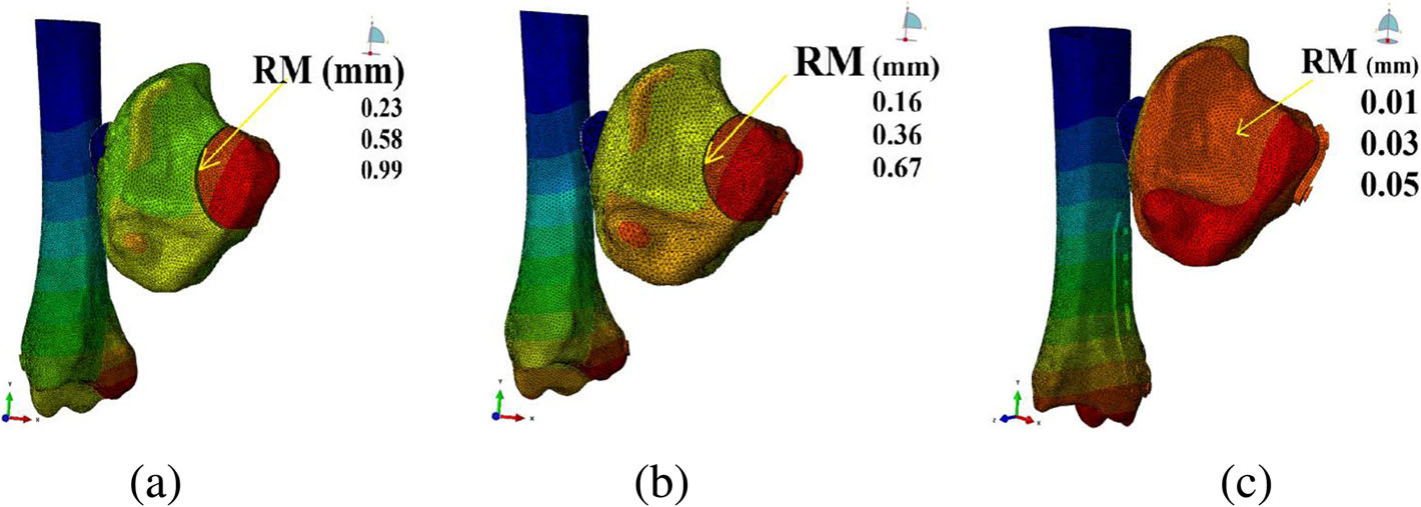
Relative micro-motion (RM) of the fracture in (**a**) AP, (**b**) PA and (**c**) posterior plate models

**Fig. 9:**
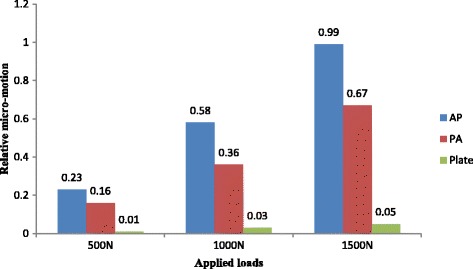
Graphical representation of Relative micro-motion (RM) of the fracture in three different fixation models

The fracture micro-motion analysis demonstrated that the mean RM in the plate group (0.03 mm) was lowest among the three models. Whereas the mean relative motions in AP and PA lag models were 0.60 mm and 0.39 mm respectively (Table 3). When magnitude of the applied load increased, the RM in the individual group was also increased. The detailed values of RM in each group are summarized in Figs. 8 and 9.

**Table 3:** Mean and standard deviation (SD) values of relative micro-motion and vertical displacement

Variables	Groups	Magnitude of applied force			Mean	SD	*P*-value*
		500 N	1000 N	1500 N			
Relative micro-motion (RM)	AP lag (1)	0.23	0.58	0.99	0.60	0.38	
	PA lag (2)	0.16	0.36	0.67	0.39	0.25	
	Post. Plate (3)	0.01	0.03	0.05	0.03	0.02	0.039 (3–1)
Vertical displacement(VD)	AP lag	0.57	1.40	1.93	1.30	0.68	
	PA lag	0.52	1.14	1.85	1.19	0.69	
	Post. Plate	0.37	0.86	1.32	0.88	0.52	

*P-value is significant at < 0.05

## Discussion

To our knowledge, this is the first computational study comparing the biomechanical efficiency of practically used three different fixation modalities (AP lag screws, PA lag screws and posterior plating) in which the force is applied in the direction of fracture mechanism. According to the findings of this finite element study, the fixation of the posterior malleolar fractures with the posterior buttress plating is most stable fixation construct than the AP and PA lag screws. This was represented by the lowest relative fracture displacement in the posterior plate group. In this study we simulated the posterior malleolar fracture of greater than 25% size of the tibial plafond. Though in recent literature there is no consensus about the indications for surgical interventions, but most of the clinicians recommended the posterior malleolar fracture of > 25% size should fix surgically [22–24]. Furthermore, biomechanical research has evaluated that the posterior malleolus plays an important role in the tibiotalar load propagation. It also prevents the posterior displacement of the talus [25, 26]. With the increasing size of the posterior fragment, the risk of posterior talar subluxation increase especially when the fracture size is greater than 25% [22].

The von Mises stress (VMS) analysis of the tibia showed the higher stress areas both in anterior and posterior side (See Fig. 5). These stress distribution patterns were different from the previously described finding [27]. The difference was due to the fact that the direction of the applied force used this study was in the opposite direction and the force was concentrated only to the fracture fragment in this analysis. Though the displacement analysis of PA and plate groups showed nearly the same amounts of displacement, but it should be noted that in PA lag model, the displacement was limited mainly to the fracture fragment to which load was applied with a negligible amount of displacement in the rest of model (Fig. 6, b). But in case of posterior plate, the displacement was also prominent in the tibial plafond (Fig. 6, c). This combined displacement of the distal tibia along with the fracture fragment signifies that the fixation strength of the plate is better than AP and PA lag screws. Due to better anchorage power of the posterior plate, it resisted the vertical displacement and the resulted displacement (Fig. 6, c) was also demonstrated in the rest of distal tibia. The model displacement comparison between AP and PA lag groups showed that the biomechanical fixation strength of PA lag screws was superior to AP lag screws, because in case of AP lag screws mainly the fracture fragment was displaced (Fig. 6, a) whereas in PA lag model a slight displacement was also observed in the tibial plafond.

In this study, the lowest vertical displacement (Y component of the displacement) was observed in the plate group which can be easily explained by the fact that the plate resisted the upward displacement more effectively than AP and PA lag screws. AP lag group with the largest VD values was the biomechanically least stable fixation construct in this study. The relative fracture micro-motion in AP lag group was also higher than other constructs.

Posterior plate with the minimum amount of RM represented that it offered the best fixation strength among the three groups. Fixation by the AP lag screws showed nearly 23 times more RM (0.23 mm) than the posterior plate (0.01 mm) and PA lag screws showed 16 times more RM (0.16 mm) as compared to the posterior plate (0.01 mm) by applying 500 N force. These inter-fragmentary motions even became more prominent when we increased the applied force to 1500 N. Clinically the less stable fixation constructs with large amounts of relative micro motion may result in implant loosening and mimics the reduction stability. The resulted loss of fracture reduction (secondary to the less stable fixation constructs) can cause successive increase in the focal contact pressure which ultimately leads to the formation of degenerative osteoarthritis [24, 28].

The previous studies have reported the outcomes of posterior plating and documented that the posterior plating is superior in achieving the fracture reduction. But these studies compared only the two fixation methods. In one of these biomechanical studies comparison was done between AP screws and posterior buttress plate and in another study, the analysis was done for PA lag screw and posterior plate [20, 29]. Here we have compared the three different fixation modalities. Our computational findings are consistent with previous biomechanical and clinical studies. In a recent biomechanical study, Bennett et al. [20] has compared the fixation strength of one-third tubular posterolateral plate and AP lag screws in the cadaveric models of posterior malleolar fractures. The results of this study advocated the less displacement using the posterior buttress plating than AP lag screws during cyclical loading. Another study published in Chinese literature, documented that the fixation of posterior malleolus with distal radius plate is more stable than PA lag screws [29]. A retrospective clinical study carried out by O’Connor et al. [30] demonstrated the improved clinical and radiographic outcomes at follow up in 27 patients treated with posterior plate compared with AP screws fixation. In our FEA though the posterior plate still remained the strongest implant for fixation of the posterior malleolus, it also showed that PA lag screws are the second most stable construct.

This work has several limitations. Firstly, soft tissues such as ligaments and syndesmosis were not simulated in this study, and only the ability of the used implants to resist the displacements and stress changes were calculated. So the stability offered by the surrounding soft tissues was ignored. But, this technical limitation affected all the groups equally and it didn’t question the validity of our findings. Secondly, it is a static simulated study and further studies are needed to explore the cyclic loading conditions.

## Conclusions

FEA of 3D posterior malleolar fracture model shows that posterior buttress plating with minimum amount of the relative fracture micro-motion and vertical displacement is biomechanically the most stable fixation implant. The second most stable construct is PA lag, whereas AP lag group with the largest RM and VD values is the least stable fixation method. Surgeons should consider the findings of this computational exploration study when selecting a fixation strategy for posterior malleolar fractures.

## Abbreviations

3D: Three dimensional
AP: Antero-posterior
CAD: Computer aided design
CT: Computed tomography
FEA: Finite element analysis
i.e.: That is
PA: Postero-anterior
PMF: Posterior malleolar fracture
RM: Relative micro-motion
VD: Vertical displacement
